# Test characteristics of shorter versions of the Alcohol, Smoking and Substance Involvement Screening Test (ASSIST) for brief screening for problematic substance use in a population sample from Israel

**DOI:** 10.1186/s13011-023-00566-7

**Published:** 2023-10-12

**Authors:** Dvora Shmulewitz, Roi Eliashar, Maor Daniel Levitin, Shaul Lev-Ran

**Affiliations:** 1 Israel Center on Addiction, 2 HaTzoran Street, Netanya, Israel; 2 Lev Hasharon Medical Center, Netanya, Israel; 3 Sackler Faculty of Medicine, Tel Aviv University, Tel Aviv, Israel

**Keywords:** ASSIST, ASSIST-FC, Problematic substance use, Test characteristics, Public health, Screening

## Abstract

**Background:**

Substance use is a leading cause of preventable morbidity and mortality worldwide. Population-wide screening for problematic substance use in primary health care may mitigate the serious health and socio-economic consequences of such use, but the standard Alcohol, Smoking and Substance Involvement Screening Test (ASSIST 3.1) may be too long for wide-scale screening. How well validated shorter versions (ASSIST-Lite, ASSIST-FC) perform in identifying those with ASSIST 3.1 problematic use in different settings is unclear.

**Methods:**

General population Jewish adults in Israel (N = 2,474) responded to an online survey that included the ASSIST 3.1 and sociodemographics. Across substances (alcohol, tobacco, cannabis, sedatives, prescription stimulants, prescription painkillers), receiver operator characteristic curve analysis determined that ASSIST-FC scores performed better than ASSIST-Lite at identifying those with problematic use, and evaluated differential ASSIST-FC performance by gender or age. Test characteristics and agreement were evaluated for binary ASSIST-FC versions, with ASSIST 3.1 problematic use as the gold standard.

**Results:**

ASSIST-FC scores showed high ability to identify ASSIST 3.1 problematic use, with minimal differences by gender or age. Binary ASSIST-FC (most substances: threshold 3+; alcohol: 5+) showed high specificity and positive predictive value, acceptable sensitivity, and good agreement.

**Conclusions:**

The ASSIST-FC, which assesses frequency of use and other’s concerns about use, appears useful for very brief screening in primary care to identify patients who may benefit from intervention. Early identification of those at-risk may prevent more severe consequences and ultimately decrease the significant costs of problematic substance use on the individual and population level.

**Supplementary Information:**

The online version contains supplementary material available at 10.1186/s13011-023-00566-7.

## Introduction

Worldwide, substance use and use disorders are leading causes of preventable morbidity and mortality [1–3] and alcohol and drug use and disorders are increasing [2, 4]. Substance use and disorders are associated with serious health, legal, social, and economic consequences [1, 3, 5]. One important strategy for combatting this major concern is population-wide screening in primary health care settings, since early identification of and intervention for problematic use can limit disorder progression and mitigate significant consequences [6, 7].

The World Health Organization developed the Alcohol, Smoking and Substance Involvement Screening Test (ASSIST 3.1), a brief, feasible, reliable, and valid screening tool for use worldwide to identify patients who could benefit from brief intervention or treatment for their substance use [6–8]. Yet, for many primary health care providers, ASSIST 3.1 is considered difficult to administer and too long to be widely adopted for general screening [9–11]. Therefore, two shorter versions that screen specific substances have been developed and validated in general population and clinical samples from a range of countries. The ASSIST-Lite [9] is short and straightforward to administer and score, as ordinal responses were replaced with binary responses, and showed good diagnostic accuracy, reliability, and validity. But the ASSIST-Lite uses different questions for different substances, may lose information by collapsing frequency questions into binary form, and was developed to identify problematic use only among those with current (past 3 months) use. The ASSIST-FC consists of two questions, frequency of use and other’s concerns about patient’s use [10]. The same questions are used across all substances and the ordinal response structure found in ASSIST 3.1 is maintained. The ASSIST-FC was shown to be reliable and valid and can identify those at risk for relapse among those without current use by querying concern about past use, but might be missing key substance specific questions. What remains unknown is which shorter version provides more information about ASSIST 3.1 and test characteristics (sensitivity, specificity, positive predictive value, negative predictive value, and chance-corrected agreement) for binary versions in different countries or settings [10].

Therefore, general population data from an online survey in Israel, a Western country, were used to assess the ASSIST 3.1, ASSIST-Lite, and ASSIST-FC across substances. A previous general population study in Israel that used similar methodology showed that prevalence of problematic substance use for alcohol, cannabis, and sedatives was similar to prevalence in the U.S. and Europe [12]. The overall goal of this study is to recommend a short form of the ASSIST 3.1 to use for quick and efficient screening of potentially problematic use in primary care, by examining the utility of shorter versions to identify those with problematic use. To that end, analysis was carried out in a stepwise fashion, with results from one step informing development of the next step. For common substances (tobacco, alcohol, cannabis, sedatives, prescription stimulants, prescription painkillers), first, we estimated the prevalence of ASSIST 3.1 problematic use. Second, we evaluated which short version (ASSIST-FC, ASSIST-Lite) performed better at identifying those with ASSIST 3.1. Third, we retained the best performing short version and tested whether its performance differed by gender or age, an important consideration for a screening test. Fourth, to provide information about binary forms of the best short version, test characteristics across various thresholds were evaluated. Finally, exploratory analysis addressed whether including craving in the best short version added information or utility, since craving is considered central to problematic use and represents a potential treatment target [13–15], and the craving question performed similarly to the concern question in the ASSIST-FC [10].

## Methods

### Sample

Using similar methodology to an epidemiological survey from 2018 [12, 16, 17], cross-sectional data were collected in April 2022 from a quasi-representative general population sample of adults living in Israel. Respondents were recruited from a demographically diverse Web panel of individuals who choose to participate in surveys, maintained by the national digital collection agency iPanel [18]. The main sample included respondents aged 18–70, as those above 70 are less likely to participate in surveys; and Hebrew speaking and Jewish, since cultural differences would require substantial methodological adaptations [19]. To construct a quasi-representative sample of the adult, Hebrew-speaking, Jewish population in Israel, a stratified sample was drawn utilizing specified quotas [20] based on age, gender, geographic area, religiosity, and education, using estimates from the 2018 survey (to allow comparisons across surveys), which were based on Central Bureau of Statistics census data. Deviations up to 2.0% from the quotas were allowed. From those eligible within strata, potential participants were selected in two ways. First, all those surveyed in 2018 who were still registered with iPanel were invited to participate. Second, of those who had not participated previously, potential participants were selected at random. When individuals agreed to participate, they were screened against the quotas, until the target number of responses was met. Strict confidentiality was maintained, as iPanel did not have access to responses, and identification information was not available to the researchers. Survey methodology is consistent with the ICC/ESOMAR International Code on Market and Social Research [18]. The Institutional Review Board of the Reichman University approved this study.

Respondents provided electronic informed consent before beginning the online survey conducted via the Qualtrics platform [21]. The survey included items related to sociodemographics, substance use, addictive behaviors, and physical and mental health. Internet surveys may be better for collecting sensitive information such as illicit substance use or other addictive behaviors [22]. Upon survey completion, participants received online gift cards worth 22 New Israeli Shekel, which was ~ 6.50 US dollars. Quality assurance was maintained by: survey by invitation to registered respondents; 3 attention checks; and identifying respondents with unexpected response patterns. Of those invited to participate (11,750), 4,948 agreed, 1,944 were excluded due to quotas, 505 did not complete the survey (135 failed attention checks, 370 dropped out), and 25 were excluded based on response patterns, for an analytical sample of 2,474.

### Measures

To assess substance use behaviors, ASSIST 3.1 was administered and scored [23]. Respondents selected substances they ever used non-medically: tobacco, alcohol, cannabis, cocaine, amphetamines, inhalants, sedatives, hallucinogens, opioids, and other substances. Culturally appropriate examples were given for each category. For each substance used, respondents reported on (1) frequency of use in the past 3 months (current use). Those with current use reported on frequency of (2) craving (strong urge to use); (3) health, social, legal, or economic problems due to use; and (4) failure to fulfill expectations due to use. Frequency response options were: never, once or twice, 1–3 times per month, 1–4 times per week, 5–7 times per week. Respondents with lifetime use were then asked (5) if anyone ever expressed concern about their use and (6) if they had trouble controlling use; responses options were no; yes, prior to the past 3 months (past); and yes, within the past 3 months (current). A separate module assessed the ASSIST 3.1 for non-medical use of prescription stimulants and prescription painkillers, rather than assuming these would be covered by the standard categories, as suggested by the US National Institute for Drug Abuse (NIDA) [24]. This study included analysis of measures for substances with > 2% prevalence of past three month use: alcohol, tobacco, cannabis, sedatives, prescription stimulants, and prescription painkillers.

For each substance, responses were weighted and a substance involvement score was calculated by summing response weights to the 6 questions, with the exception of tobacco, where failure to fulfill expectations was excluded. As done previously [12], except for alcohol, a binary variable (problematic use) was defined as a score of 4 or more (4+), combining moderate risk (4–26) and high risk (27+) levels, which correlated with substance abuse and dependence [6] that are now considered to indicate a combined substance use disorder [25]. For alcohol, 10 + was used as the cutoff for problematic use. In sensitivity analysis, 8 + was used to indicate problematic use for cannabis, which may have better sensitivity and specificity [26], probably due to changing use practices.

The ASSIST-Lite [9] contains items that have binary yes/no responses, adapted from the ASSIST 3.1 questions (listed above). These items were scored based on the ASSIST 3.1 responses. Item 1, any current use, was positive for those who responded at least “once or twice” for frequency of current use. Item 2, using at least weekly, was positive for those who responded at least “1–4 times per week” for frequency of current use. Item 3, craving at least weekly, was positive for those who responded at least “1–4 times per week” for frequency of craving. Items 4 and 5, current concern about use and current difficulty controlling use, were positive for those who responded “yes, within the past three months” to those questions. For different substances, different items were included: for cannabis and sedatives, items 1, 3, and 4; for prescription stimulants, items 1, 2, and 4; for prescription painkillers, items 1, 4, and 5; and for alcohol, items 1, 4, 5, and any binge use (4 or more drinks per occasion), as assessed in the Alcohol Use Disorders Identification Test. For tobacco, the ASSIST-Lite included items from the Fagerstrom Test for Nicotine Dependence which was not assessed in this survey. Instead, only item 1 was used, as suggested in the modified ASSIST-Lite developed by the UK National Health Services [27]. For each substance, the relevant items were summed to create ASSIST-Lite scores.

The ASSIST-FC [10] contains two ASSIST 3.1 questions, frequency of use and other’s concern about use. For each substance, weighted responses for those two ASSIST 3.1 items were summed to create ASSIST-FC scores. As planned, since ASSIST-FC scores performed better than ASSIST-Lite scores (see **Test performance**, below), binary ASSIST-FC versions were constructed with thresholds starting from 2+ (corresponding to moderate and high risk levels; 6 + for alcohol), and including 3 + and 4+ (alcohol, 5 + and 7+).

To explore the inclusion of craving, for each substance, the ASSIST-FCr score was calculated by summing the weighted responses for frequency of use and craving, and binary versions were constructed with thresholds from 2 to 4 for all except alcohol (7–9), with additional thresholds for cannabis (5,6) to match ASSIST 3.1 with the 8 + threshold. The ASSIST-FCCr score was calculated by summing the weighted responses for frequency of use and craving, and other’s concern, and binary versions were constructed with thresholds from 3 to 5 for all except alcohol (8–10), and additional thresholds for cannabis (6,7).

Sociodemographic moderators included gender (men; women) and age (18–25; 26–34; 35–49; 50–70).

### Analysis

Sample descriptives were calculated for sociodemographic variables, substance use, and ASSIST 3.1 problematic use (moderate or high risk levels). Analysis was planned such that results from one step would inform the next step, e.g., the most informative of the two shorter versions would be kept for additional analyses. For each substance, receiver operator characteristic (ROC) curve analysis was used to assess the area under the curve (AUC), which evaluates the ability of the ASSIST-Lite and ASSIST-FC scores to identify those with ASSIST 3.1 problematic use. AUC scores ≥ 0.9 are considered excellent, and between 0.8 and 0.89 are considered good [28]. To determine which version performed better, AUC values for ASSIST-Lite and ASSIST-FC were considered to differ if the 95% confidence intervals (CI) were non-overlapping. For the better scores (ASSIST-FC), to see if scores showed differential performance by gender or age, AUC values were compared, by taking the difference between values for men and women, and for each age group and the control group (18–25). AUC values were considered different if the 95% CI for the difference did not overlap with 0.

Since the goal was to determine how well the short forms could identify those with ASSIST 3.1 problematic use (“gold standard”), for each substance, test characteristics for the ASSIST-FC binary variables (tests) were calculated from the true positives (TP; yes for ASSIST-FC and ASSIST 3.1), false negatives (FN; no for ASSIST-FC and yes for ASSIST 3.1), false positives (FP; yes for ASSIST-FC and no for ASSIST 3.1) and true negatives (TN; no for ASSIST-FC and ASSIST 3.1). Sensitivity, ability to correctly classify an individual as having the outcome of interest, measures the proportion of those with problematic use correctly identified by the test [TP/TP + FN]. Specificity, ability to correctly classify an individual as not having the outcome of interest, measures the proportion of those without problematic use correctly identified [TN/FP + TN]. Positive predictive value (PPV) is the proportion of those with a positive test who have problematic use [TP/TP + FP], and negative predictive value (NPV) is the proportion of those with a negative test who don’t have problematic use [TN/TN + FN]. Since the study goal is to identify tests to efficiently screen for patients who would benefit from brief intervention or referral to specialist treatment, where they would score high on further tests, e.g., ASSIST 3.1., tests with high specificity would be favored, where a positive score rules in problematic use, because without problematic use those would have tested negative; similarly, high PPV, indicating fewer false positives, would be favored.

Chance corrected agreement (kappa) was evaluated for ASSIST-FC binary versions and ASSIST 3.1, with kappa > = 0.61 considered good [9, 29].

To provide information about intervention needs, among those with ASSIST-FC binary versions, the percent with current use was calculated.

#### Exploratory analysis: including craving

ROC curve analysis compared ASSIST-FC, ASSIST-FCr, and ASSIST-FCCr scores. Test characteristics and agreement were assessed for binary versions of ASSIST-FCr and ASSIST-FCCr with ASSIST 3.1 problematic use, and were compared to estimates for ASSIST-FC; estimates with non-overlapping CI were considered to differ. Among those with binary ASSIST-FCCr, the percent with current use was calculated; all those with non-zero scores on ASSIST-FCr have current use.

Analysis was conducted using SPSS software version 28 [30].

## Results

### Descriptives

About half the sample were women, attained post high school academic education; about 40% were aged 18–34, not married; and almost three-quarters had any employment (Table 1). Current substance use ranged from 76.0% (alcohol) to 3.4% (non-medical use of prescription painkillers), and problematic use ranged from 36.9% (tobacco) to 2.6% (prescription painkillers), with 23.1% showing problematic use for any drug or alcohol (Table 1).

**Table 1:** Sample descriptives (N = 2,474)

	n	Percent (%)
**Gender**		
Men	1,233	49.8
Women	1,218	49.2
Other	23	0.9
**Age**		
18–25	452	18.3
26–34	590	23.8
35–49	709	28.7
50–70	723	29.2
**Educational attainment**		
High school, non-matriculation	194	7.8
High school, matriculation	499	20.2
Post-high school, technological	530	21.4
Post-high school, academic	1,251	50.6
**Marital status**		
Married	1,419	57.4
Not married	1,055	42.6
**Employment status**		
Any job	1,785	72.2
No job	689	27.8
**Religiosity**		
Secular	1,287	52.0
Traditional	766	31.0
National Religious	349	14.1
Ultra-Orthodox	72	2.9
**Residency**		
Jerusalem Area	276	11.2
Tel Aviv and Central area	1,012	40.9
North	643	26.0
South	543	21.9
**Ethnicity**		
Middle Eastern/North African	970	39.2
European, not Former Soviet Union	798	32.3
Former Soviet Union	237	9.6
Mixed	407	16.5
Other	62	2.5
**Past three months substance use**		
Tobacco	868	35.1
Alcohol	1,881	76.0
Cannabis	356	14.4
Sedatives	113	4.6
Prescription stimulants	124	5.0
Prescription painkillers	83	3.4
Other drugs^a^	58	2.3
**ASSIST 3.1 Problematic use** ^**b**^		
Tobacco	914	36.9
Alcohol	325	13.1
Cannabis	269	10.9
Cannabis, problematic use defined as 8**+**	168	6.8
Sedatives	96	3.9
Prescription stimulants	94	3.8
Prescription painkillers	65	2.6
Other drugs^a^	50	2.3
Any drug	354	14.3
Any drug or alcohol	571	23.1

a cocaine, amphetamines, hallucinogens, inhalants, or opioids

b ASSIST 3.1 score 4+, indicating moderate or high risk levels

### Test performance

ASSIST-FC scores showed excellent ability to identify those with problematic use (AUC > 0.9). AUC values were higher for ASSIST-FC than ASSIST-Lite scores, with significantly better performance at identifying ASSIST 3.1 problematic use for tobacco, alcohol and cannabis (Table 2). As planned, the ASSIST-FC measures were selected for further analyses. No significant differences in test performance (AUC) were observed by gender (Table 3). Minimal differences were observed by age: cannabis and prescription stimulants scores performed slightly better and sedatives score performed slightly worse for ages 50–70 than ages 18–25; and prescription stimulants score performed slightly better for ages 35–49 than ages 18–25 (Table 4).

**Table 2:** Performance of ASSIST-FC and ASSIST-Lite scores, for identifying problematic use^a^

	*Main analysis*		*Additional analyses with craving*	
	ASSIST-Lite	ASSIST-FC	ASSIST-FCr	ASSIST-FCCr
**Substance**	Area under the curve (AUC) (95% confidence interval)			
Tobacco	0.86 (0.84, 0.88)	0.96 (0.95, 0.97)^b^	0.92 (0.91, 0.93)	0.96 (0.95, 0.97)
Alcohol	0.77 (0.74, 0.80)	0.86 (0.84, 0.89)^b^	0.85 (0.83, 0.87)	0.92 (0.91, 0.94)^c^
Cannabis	0.89 (0.86, 0.91)	0.94 (0.92, 0.95)^b^	0.94 (0.92, 0.96)	0.99 (0.98, 0.99)^c^
Cannabis (8 + threshold)	0.91 (0.89, 0.932)	0.94 (0.925, 0.96)	0.95 (0.94, 0.97)	0.98 (0.97, 0.99)^c^
Sedatives	0.89 (0.85, 0.94)	0.95 (0.92, 0.975)	0.96 (0.923, 0.94)	0.99 (0.977, 1.00)^c^
Prescription stimulants	0.87 (0.82, 0.92)	0.94 (0.91, 0.962)	0.91 (0.86, 0.95)	0.98 (0.963, 0.99)^c^
Prescription painkillers	0.84 (0.77, 0.90)	0.92 (0.88, 0.96)	0.90 (0.85, 0.96)	0.97 (0.95, 1.00)

FC = frequency and concern; FCr = frequency and craving; FCCr = frequency, concern, and craving

a ASSIST 3.1 moderate or high risk levels

Area under the curve (AUC) differ significantly where the 95% confidence intervals do not overlap: ^b^ASSIST-FC scores showed significantly higher AUC than ASSIST-Lite scores; ^c^ASSIST-FCCr scores showed significantly higher AUC than ASSIST-FC scores

**Table 3:** Performance of ASSIST-FC scores by gender

	AUC (95% CI)		
**Substance**	Men	Women	**Difference in AUC (95% CI)** ^**a**^
Tobacco	0.96 (0.95, 0.97)	0.96 (0.95, 0.97)	0.00 (-0.01, 0.02)
Alcohol	0.85 (0.82, 0.88)	0.87 (0.83, 0.91)	-0.01 (-0.06, 0.04)
Cannabis	0.93 (0.92, 0.95)	0.94 (0.92, 0.96)	-0.01 (-0.04, 0.02)
Cannabis (8 + threshold)	0.94 (0.91, 0.96)	0.95 (0.92, 0.97)	-0.01 (-0.05, 0.03)
Sedatives	0.95 (0.91, 0.99)	0.95 (0.90, 0.99)	0.01 (-0.05, 0.06)
Prescription stimulants	0.91 (0.87, 0.96)	0.96 (0.93, 0.99)	-0.04 (-0.10, 0.01)
Prescription painkillers	0.93 (0.88, 0.98)	0.92 (0.86, 0.98)	0.01 (-0.07, 0.09)

FC = frequency and concern; AUC = area under the curve; CI = confidence interval

a AUC differ significantly where the 95% CI around the difference does not include 0; no differences were significant

**Table 4:** Performance of ASSIST-FC scores by age

	AUC (95% CI)				Difference in AUC (95% CI)^a^		
**Substance**	18–25	26–34	35–49	50–70	26–34 vs. 18–25	35–49 vs. 18–25	50–70 vs. 18–25
Tobacco	0.96 (0.93, 0.98)	0.97 (0.96, 0.98)	0.96 (0.94, 0.98)	0.96 (0.94, 0.97)	0.01 (-0.01, 0.04)	0.00 (-0.03, 0.03)	0.00 (-0.03, 0.03)
Alcohol	0.88 (0.83, 0.92)	0.83 (0.78, 0.87)	0.86 (0.82, 0.90)	0.89 (0.84, 0.94)	-0.05 (-0.12, 0.01)	-0.02 (-0.08, 0.05)	0.01 (-0.06, 0.08)
Cannabis	0.91 (0.87, 0.96)	0.93 (0.90, 0.96)	0.93 (0.91, 0.96)	0.98 (0.96, 1.00)	0.02 (-0.04, 0.07)	0.02 (-0.03, 0.08)	*0.06 (0.01, 0.11)*
Cannabis (8 + threshold)	0.92 (0.87, 0.97)	0.94 (0.91, 0.97)	0.95 (0.92, 0.98)	0.95 (0.91, 0.99)	0.02 (-0.04, 0.08)	0.03 (-0.03, 0.09)	0.03 (-0.04, 0.09)
Sedatives	0.99 (0.97, 1.02)	0.96 (0.90, 1.02)	0.96 (0.90, 1.01)	0.94 (0.89, 0.98)	-0.03 (-0.10, 0.29)	-0.04 (-0.09, 0.24)	*-0.05 (-0.11, -0.00)*
Prescription stimulants	0.89 (0.81, 0.96)	0.92 (0.86, 0.98)	0.97 (0.94, 1.01)	0.98 (0.93, 1.02)	0.03 (-0.06, 0.13)	*0.09 (0.01, 0.17)*	*0.09 (0.00, 0.18)*
Prescription painkillers	0.84 (0.67, 1.01)	0.95 (0.87, 1.03)	0.91 (0.83, 0.98)	0.95 (0.89, 1.01)	0.11 (-0.08, 0.30)	0.07 (-0.12, 0.26)	0.11 (-0.07, 0.29)

FC = frequency and concern; AUC = area under the curve; CI = confidence interval

a AUC differ significantly where the 95% CI around the difference does not include 0; significant differences are shown in italics

### Test characteristics and agreement

Recommendations are that screening tests should be highly specific with high PPV, to limit additional testing to those who are most likely to be true positives, with good agreement between the test and gold standard [6, 9]. Additionally, sensitivity should be acceptable, to limit false negatives. For all substances, specificity/PPV increased as the threshold increased, and there existed a threshold with high specificity/PPV, acceptable sensitivity, and good kappa, except alcohol (Table 5). Generally, the middle threshold showed similar prevalence to ASSIST 3.1 problematic use (Table 5). Thus, for all substances but alcohol, the middle threshold (3+) appeared to be a reasonable choice. For alcohol, the best option appeared to be 5+. For cannabis defining moderate as 8–26, 4 + appeared appropriate.

**Table 5:** Test characteristics for ASSIST-FC thresholds, as compared to ASSIST 3.1 problematic use

Substance (prevalence of ASSIST 3.1)	N	Prevalence %	Sensitivity %	Specificity %	Positive predictive value %	Negative predictive value %	Agreement (kappa)
*Tobacco (36.9%)*							
2+	1,046	42.3	99.5 (98.7, 99.8)	91.2 (89.7, 92.6)	86.9 (84.7, 88.9)	99.6 (99.2, 99.9)	0.88 (0.86, 0.90)
3+	885	35.8	88.5 (86.3, 90.5)	95.1 (93.9, 96.1)	91.4 (89.4, 93.2)	93.4 (92.1, 94.6)	0.84 (0.82, 0.86)
4+	641	25.9	70.1 (67.0, 73.1)	100 (99.8, 100)	100 (99.4, 100)	85.1 (83.4, 86.7)	0.75 (0.72, 0.78)
*Alcohol (13.1%)*							
5+	323	13.1	64.9 (59.5, 70.1)	94.8 (93.8, 95.7)	65.3 (59.9, 70.5)	94.7 (93.7, 95.6)	0.60 (0.55, 0.65)
6+	291	11.8	60.0 (54.4. 65.4)	95.5 (94.6, 96.4)	67.0 (61.3, 72.4)	94.0 (93.4, 95.0)	0.58 (0.53, 0.63)
7+	152	6.1	39.7 (34.3, 45.2)	98.9 (98.4, 99.3)	84.9 (78.2, 90.2)	91.6 (90.4, 92.7)	0.50 (0.44, 0.55)
*Cannabis (10.9%)*							
2+	408	16.5	100 (98.6, 100)	93.7 (92.6, 94.7)	65.9 (61.1, 70.5)	100 (99.8, 100)	0.76 (0.73, 0.80)
3+	261	10.5	75.1 (69.5, 80.1)	97.3 (96.9, 98.0)	77.4 (71.8, 82.3)	97.0 (96.2, 97.6)	0.73 (0.69, 0.78)
4+	148	6.0	55.0 (48.9, 61.1)	100 (99.8, 100)	100 (97.5, 100)	94.8 (93.8, 95.7)	0.69 (0.63, 0.73)
*Cannabis (threshold 8+; 6.8%)*							
2+	408	16.5	100 (97.8, 100)	89.6 (88.3, 90.8)	41.2 (36.4, 46.1)	100 (99.8, 100)	0.54 (0.49, 0.59)
3+	261	10.5	87.5 (81.5, 92.1)	95.1 (94.1, 95.9)	56.3 (50.1, 62.4)	99.1 (98.6, 99.4)	0.66 (0.60, 0.71)
4+	148	6.0	76.8 (69.7, 82.9)	99.2 (98.7, 99.5)	87.2 (80.7, 92.1)	98.3 (97.7, 98.8)	0.80 (0.75, 0.85)
*Sedatives (3.9%)*							
2+	122	4.9	99.0 (94.3, 100)	98.9 (98.4, 99.3)	77.9 (68.5, 84.9)	100 (99.8, 100)	0.87 (0.81, 0.91)
3+	85	3.4	79.2 (69.7, 86.8)	99.6 (99.3, 99.8)	89.4 (80.8, 95.0)	99.2 (98.7, 99.5)	0.83 (0.77, 0.89)
4+	52	2.1	54.2 (43.7, 64.4)	100 (99.8, 100)	100 (93.2, 100)	98.2 (97.6, 98.7)	0.69 (0.60, 0.77)
*Prescription stimulants (3.8%)*							
2+	144	5.8	100 (96.2, 100)	97.9 (97.2, 98.4)	65.3 (56.9, 73.0)	100 (99.8, 100)	0.78 (0.72, 0.84)
3+	93	3.8	77.7 (67.9, 85.6)	99.2 (98.7, 99.5)	78.5 (68.8, 86.3)	99.1 (98.7, 99.5)	0.77 (0.70, 0.83)
4+	55	2.2	58.5 (47.9, 68.6)	100 (99.8, 100)	100 (93.5, 100)	98.4 (97.8, 98.9)	0.73 (0.64, 0.81)
*Prescription painkillers (2.6%)*							
2+	93	3.8	98.5 (91.7, 100)	98.8 (98.3, 99.2)	68.8 (58.4, 78.0)	100 (99.8, 100)	0.80 (0.74, 0.87)
3+	47	1.9	64.6 (51.8. 76.1)	99.8 (99.5, 99.9)	89.4 (76.9, 96.5)	99.1 (98.6, 99.4)	0.74 (0.64, 0.83)
4+	31	1.3	47.7 (35.1, 60.5)	100 (99.8, 100)	100 (88.8, 100)	98.6 (98.1, 99.0)	0.64 (0.52, 0.75)

FC = frequency and concern

### Current use among those with problematic use

For alcohol-FC 5+, all those positive currently drank alcohol. For other substances, of those positive for ASSIST-FC 3+, the prevalence of those without current use ranged from 10.6% (sedatives) to 21.5% (prescription stimulants) (Supplementary Table 1 found in Additional file 1).

### Including craving

ASSIST-FCr scores were not significantly better at identifying ASSIST 3.1 problematic use than ASSIST-FC scores (Table 2). Note that ASSIST-FCr only provides information among those with current use. For tobacco, cannabis, and sedatives, ASSIST-FCr 3 + showed greater specificity or PPV than ASSIST-FC 3+; for cannabis, agreement was also greater (Supplementary Table 2). ASSIST-FCCr scores generally showed higher ability than ASSIST-FC scores and performed significantly better for alcohol, cannabis, sedatives, and prescription stimulants (Table 2). Comparing ASSIST-FCCr 4 + to ASSIST-FC 3+, specificity and PPV were greater for tobacco; all measures were greater for cannabis; sensitivity, PPV, and kappa were greater for sedatives; and specificity, PPV, and kappa were greater for prescription stimulants (Supplementary Table 2 found in Additional file 1). No differences were observed for cannabis, 8 + threshold. For alcohol, ASSIST-FCCr 9 + appears to be reasonable. For ASSIST-FCCr 4+ (alcohol, 9+), almost all with problematic use had current use (Supplementary Table 3 found in Additional file 1).

## Discussion

Using data from an epidemiological survey of a general population sample of Hebrew-speaking Jews living in Israel, a Western country, the prevalence of problematic substance use was reported and shorter ASSIST versions were compared to develop recommendations for a brief screening test for efficient use in primary care.

In terms of prevalence, there are two main considerations. First, for substances reported on in the previous survey from Israel (alcohol, cannabis, and sedatives) [12], prevalence of ASSIST 3.1 problematic use either descriptively increased or remained similar; for additional substances, prevalence was high for tobacco and non-negligible for prescription stimulants and painkillers, suggesting that problematic use is a pressing and possibly increasing public health concern in Israel, as worldwide [1–3]. Second, although the ASSIST 3.1 queries non-medical use of amphetamines, opioids, and other drugs, which should cover prescription stimulants and opiate painkillers, very few respondents screened into those sections. More respondents screened in when asked specifically about prescription stimulants and painkillers in a later module. This suggests that when the ASSIST is self-administered, specific categories of prescription medications should be included in the list of substances, as in the modified NIDA version [24].

Study results support several recommendations for a brief screening test. Lifetime use should be asked regarding general substance categories, specifically listing relevant prescription medications, with very rare substances in the “other” category. This may differ by country but would save time by limiting irrelevant questions. The ASSIST-FC, which queries frequency of use and other’s concerns about use, performed well at identifying those with ASSIST 3.1 problematic use, with few differences by gender or age. These two items show face validity for identifying those who may require clinical interventions. The ASSIST-FC can be used in count form (with higher values indicating more severe problems) or in binary form, with a threshold of 3+ (alcohol, 5+) generally providing prevalence similar to ASSIST 3.1, high specificity/PPV, acceptable sensitivity, and good agreement.

Those identified as having problematic use fall into two categories. The majority currently use the substance; these individuals can receive brief intervention and/or referral to specialist care for further testing and treatment, based on the health care provider’s discretion. Others had past concerns about use but did not use currently; these individuals can receive short validation of their success in abstaining and recommendation to return to the medical practitioner if abstinence becomes difficult. More generally, whether those without current use should be considered to have problematic use warrants further study. Paying extra attention to such individuals may mitigate their increased risk for relapse, but including them may lead to possibly misleading prevalence rates of problematic use, since they are not actually using.

There are additional points to consider. The ASSIST-FC did not perform well for alcohol, similar to the original ASSIST-FC study, which suggests replacing frequency of any use with frequency of binge drinking [10]. In exploratory analysis, the ASSIST-FC score with binge drinking (AUC = 0.84; 95% CI = 0.82, 0.87) did not perform better than the score with any use. Instead, perhaps craving should be included for alcohol, since the ASSIST-FCCr score performed better than ASSIST-FC for alcohol and the 9 + ASSIST-FCCr binary version showed acceptable test characteristics. The ASSIST-FCCr measures also showed better performance for other substances. Although the original ASSIST-FC did not recommend including craving since there were only small increases in test performance with craving [10], here significant improvements in test characteristics were observed. While further studies are warranted to validate the ASSIST-FCCr across substances, since many clinicians consider craving to be central to addiction and treating craving may lead to successful reduction in use, health care providers can consider including craving in the test. Additionally, the ASSIST-FC threshold could be increased to 4 + for cannabis as use becomes more normative. Last, the study aim was to recommend a shorter version of the ASSIST 3.1, so ASSIST 3.1 was the “gold standard”. But the ultimate goal is to develop efficient screening tests with strong ability to predict clinical diagnoses and treatment-related outcomes. Additional studies should assess the validity of the shorter versions (ASSIST-FC, ASSIST-FCCr) and determine their predictive ability, cross-culturally.

Study limitations are noted. First, there may be selection bias since respondents were limited to those willing and able to participate in the online survey, but using quotas enabled collection of a sample that can be considered relatively representative of the adult, Jewish, Hebrew-speaking population of Israel, in terms of key sociodemographic factors (age, gender, geographic area, religiosity, and education) [12, 16, 17]. Nevertheless, those of lower socioeconomic status may be less likely to participate in internet survey panels and may be more likely to use substances and have higher rates of problematic use; thus, prevalence may be underestimated. Second, the analyzed sample was missing key sectors of the Israeli population that would need methodological adaptations, e.g., those less likely to complete online surveys (older than 70, Ultra-Orthodox) or with cultural differences (Israeli-Arabs). Pilot studies within those groups have been conducted to determine how to collect more representative samples of the entire population in future studies. Third, non-Hebrew speakers were excluded, but > 90% of Jews in Israel have mastery of Hebrew [31]. Fourth, other biases may exist. Responses were subject to recall bias, which may be limited by the short timeframe for current use (three months). Responses were dependent on participant’s understanding of the questions, but standard, validated screening instruments were used. There may be hesitancy on reporting possibly stigmatized or illegal behaviors; this should be mitigated by the confidential online platform [22]. Fifth, no analyses were done for substances less prevalent in Israel. Sixth, the ASSIST-Lite could not be assessed for tobacco but a proxy measure (any current use) was used instead [27]. Seventh, the gold standard was the ASSIST 3.1; how well the ASSIST-FC performs as compared to clinical diagnoses should be determined in future studies. Yet, this study evaluated the shorter ASSIST versions in the general population, which may be more informative for primary care settings than the original studies, which included clinical samples [9, 10].

## Conclusion

This study provides essential information about the ASSIST-FC, which can be used to screen for problematic use in primary care settings, to identify quickly and efficiently those who can benefit from brief intervention or to recommend further testing and referral to treatment, for the ultimate goal of decreasing the significant health and socio-economic costs of substance use and disorders on both the individual and population level.

### Electronic supplementary material

Below is the link to the electronic supplementary material.

**Additional file 1** is a Word document (Additional_file_1.docx) which includes three supplementary tables.

## Data Availability

The datasets used during the current study are available from the corresponding author on reasonable request.

## Abbreviations

ASSIST 3.1: Alcohol, Smoking and Substance Involvement Screening Test
ASSIST-FC: ASSIST – frequency and concern
ASSIST-FCr: ASSIST – frequency and craving
ASSIST-FCCr: ASSIST – frequency, concern, and craving
AUC: Area under the curve
CI: Confidence interval
FN: False negative
FP: False positive
NIDA: National Institute for Drug Abuse (US)
NPV: Negative predictive value
PPV: Positive predictive value
ROC: Receiver operator characteristic
TN: True negative
TP: True positive
