# Supplementary material for: Test characteristics of shorter versions of the Alcohol, Smoking and Substance Involvement Screening Test (ASSIST) for brief screening for problematic substance use in a population sample from Israel
Source: Subst Abuse Treat Prev Policy. 2023 Oct 12;18:58. doi: 10.1186/s13011-023-00566-7 (PMC10571312; doi:10.1186/s13011-023-00566-7)
Supplement: Supplementary file 1 — Additional file 1 is a Word document (Additional_file_1.docx) which includes three supplementary tables. [file 13011_2023_566_MOESM1_ESM.docx]

**Supplementary Table 1:** Prevalence of current use among those with ASSIST 3.1 and ASSIST-FC binary versions

|  | **ASSIST 3.1** | | | | | **ASSIST-FC, 2+** | | | | | **ASSIST-FC, 3+** | | | | | **ASSIST-FC, 4+** | | | | |
| --- | --- | --- | --- | --- | --- | --- | --- | --- | --- | --- | --- | --- | --- | --- | --- | --- | --- | --- | --- | --- |
|  |  | No current use | | With current use | |  | No current use | | With current use | |  | No current use | | With current use | |  | No current use | | With current use | |
|  | N with disorder | n | % | n | % | N with disorder | n | % | n | % | N with disorder | n | % | n | % | N with disorder | n | % | n | % |
| Tobacco | 914 | 125 | 13.7 | 789 | 86.3 | 1,046 | 178 | 17.0 | 868 | 83.0 | 885 | 178 | 20.1 | 707 | 79.9 | 641 | 4 | 0.6 | 637 | 99.4 |
| Alcohol^a^ | 325 | 0 | 0 | 325 | 100 | 323 | 0 | 0 | 323 | 100 | 291 | 0 | 0 | 291 | 100 | 152 | 0 | 0 | 152 | 100 |
| Cannabis | 269 | 21 | 7.8 | 248 | 92.2 | 408 | 52 | 12.7 | 356 | 87.3 | 261 | 52 | 19.9 | 209 | 80.1 | 148 | 3 | 2.0 | 145 | 98.0 |
| Sedatives | 96 | 6 | 6.3 | 90 | 93.6 | 122 | 9 | 7.4 | 113 | 92.6 | 85 | 9 | 10.6 | 76 | 89.4 | 52 | 0 | 0 | 52 | 100 |
| Prescription stimulants | 94 | 10 | 10.6 | 84 | 89.4 | 144 | 20 | 13.9 | 124 | 86.1 | 93 | 20 | 21.5 | 73 | 78.5 | 55 | 0 | 0 | 55 | 100 |
| Prescription painkillers | 65 | 8 | 12.3 | 57 | 87.7 | 93 | 10 | 10.8 | 83 | 89.2 | 47 | 10 | 21.3 | 37 | 78.7 | 31 | 1 | 3.2 | 30 | 96.8 |

ASSIST = Alcohol, Smoking and Substance Involvement Screening Test; FC=frequency and concern

a thresholds for ASSIST-FC are 5+, 6+, 7+

**Supplementary Table 2:** Test characteristics for ASSIST-FCr and ASSIST-FCCr thresholds, as compared to ASSIST 3.1 problematic use (moderate/high risk levels)

| **Substance (prevalence ASSIST 3.1)** | N | Prevalence % | Sensitivity % | Specificity % | Positive predictive value % | Negative predictive value % | Agreement (kappa) |
| --- | --- | --- | --- | --- | --- | --- | --- |
| ***Tobacco (36.9%)*** |  |  |  |  |  |  |  |
| FC 3+ | 885 | 35.8 | 88.5 (86.3, 90.5) | 95.1 (93.9, 96.1) | 91.4 (89.4, 93.2) | 93.4 (92.1, 94.6) | 0.84 (0.82, 0.86) |
| FCr |  |  |  |  |  |  |  |
| 2+ | 868 | 35.1 | 86.3 (83.9, 88.5) | 94.9 (93.7, 96.0) | 90.0 (88.8, 92.7) | 92.2 (90.8, 93.5) | 0.82 (0.80, 0.84) |
| 3+ | 787 | 31.8 | 84.1 (81.6, 86.4) | 98.8 (98.2, 99.3) | 97.7 (96.4, 98.6) | 91.4 (90.0, 92.7) | 0.85 (0.83, 0.88) |
| 4+ | 765 | 30.9 | 83.7 (81.1, 86.0) | 100 (99.8, 100) | 100 (99.5, 100) | 91.3 (89.8, 92.6) | 0.87 (0.84, 0.89) |
| FCCr |  |  |  |  |  |  |  |
| 3+ | 977 | 39.5 | 98.6 (97.6, 99.2) | 95.1 (93.9, 96.1) | 92.2 (90.4, 93.8) | 99.1 (98.5, 99.5) | 0.92 (0.91, 0.94) |
| 4+ | 785 | 31.7 | 85.9 (83.5, 88.1) | 100 (99.8, 100) | 100 (99.5, 100) | 92.4 (91.0, 93.6) | 0.89 (0.86, 0.90) |
| 5+ | 776 | 31.4 | 84.9 (82.4, 87.2) | 100 (99.8, 100) | 100 (99.5, 100) | 91.9 (90.5, 93.1) | 0.88 (0.86, 0.89) |
| ***Alcohol (13.1%)*** |  |  |  |  |  |  |  |
| FC 5+ | 323 | 13.1 | 64.9 (59.5, 70.1) | 94.8 (93.8, 95.7) | 65.3 (59.9, 70.5) | 94.7 (93.7, 95.6) | 0.60 (0.55, 0.65) |
| FCr |  |  |  |  |  |  |  |
| 7+ | 616 | 24.9 | 75.4 (70.3, 80.0) | 82.7 (81.1, 84.3) | 39.8 (35.9, 43.8) | 45.7 (94.7, 96.6) | 0.42 (0.38,0.46) |
| 8+ | 327 | 13.2 | 54.2 (48.6, 59.7) | 93.0 (91.8, 94.0) | 53.8 (48.3, 54.3) | 93.1 (91.9, 94.1) | 0.47 (0.42, 0.52) |
| 9+ | 215 | 8.7 | 37.5 (32.3, 43.1) | 95.7 (94.7, 96.5) | 56.7 (49.8, 63.5) | 91.0 (89.8, 92.2) | 0.39 (0.33, 0.45) |
| FCCr |  |  |  |  |  |  |  |
| 8+ | 442 | 17.9 | 76.6 (71.6, 81.1) | 91.0 (89.7, 92.2) | 56.3 (51.6, 61.0) | 96.3 (95.3, 97.6) | 0.59 (0.54, 0.63) |
| 9+ | 353 | 14.3 | 68.9 (63.6, 73.9) | 94.0 (92.2, 95.0) | 63.5 (58.2, 68.5) | 95.2 (94.2, 96.1) | 0.61 (0.56, 0.65) |
| 10+ | 204 | 8.2 | 53.8 (48.3, 59.4) | 98.7 (98.1, 99.1) | 85.8 (80.2, 90.3) | 93.4 (92.3, 94.4) | 0.62 (0.57, 0.67) |
| ***Cannabis (10.9%)*** |  |  |  |  |  |  |  |
| FC 3+ | 261 | 10.5 | 75.1 (69.5, 80.1) | 97.3 (96.9, 98.0) | 77.4 (71.8, 82.3) | 97.0 (96.2, 97.6) | 0.73 (0.69, 0.78) |
| FCr |  |  |  |  |  |  |  |
| 2+ | 356 | 14.4 | 92.2 (88.3, 95.1) | 95.1 (94.1, 96.0) | 69.7 (64.6, 74.4) | 99.0 (98.5, 99.4) | 0.76 (0.73, 0.80) |
| 3+ | 254 | 10.3 | 84.0 (79.1, 88.2) | 98.7 (98.2, 99.2) | 89.0 (84.5, 92.5) | 98.1 (97.4, 98.6) | 0.85 (0.82, 0.89) |
| 4+ | 218 | 8.8 | 81.0 (75.8, 88.5) | 100 (99.8, 100) | 100 (98.3, 100) | 97.7 (97.0, 98.3) | 0.88 (0.85, 0.91) |
| FCCr |  |  |  |  |  |  |  |
| 3+ | 319 | 12.9 | 96.7 (93.7, 98.5) | 97.3 (96.9, 98.0) | 81.5 (76.8, 85.6) | 99.6 (99.2, 99.8) | 0.87 (0.84, 0.90) |
| 4+ | 239 | 9.7 | 88.8 (84.5, 92.3) | 100 (99.8, 100) | 100 (98.5, 100) | 98.7 (98.1, 99.1) | 0.93 (0.91, 0.96) |
| 5+ | 235 | 9.5 | 87.4 (83.8, 91.1) | 100 (99.8, 100) | 100 (98.4, 100) | 98.5 (97.9, 98.9) | 0.93 (0.90, 0.95) |
| ***Cannabis (threshold 8+; 6.8%)*** | |  |  |  |  |  |  |
| FC 4+ | 148 | 6.0 | 76.8 (69.7, 82.9) | 99.2 (98.7, 99.5) | 87.2 (80.7, 92.1) | 98.3 (97.7, 98.8) | 0.80 (0.75, 0.85) |
| FCr |  |  |  |  |  |  |  |
| 4+ | 218 | 8.8 | 88.1 (82.2, 92.6) | 97.0 (96.2, 97.6) | 67.9 (61.3, 74.0) | 99.1 (98.6, 99.5) | 0.75 (0.70, 0.79) |
| 5+ | 214 | 8.6 | 88.1 (82.2, 92.6) | 97.1 (96.4, 97.8) | 63.0 (56.5, 69.2) | 99.1 (98.6, 99.5) | 0.76 (0.71, 0.80) |
| 6+ | 144 | 5.8 | 69.0 (61.5, 75.9) | 98.8 (98.2, 99.2) | 80.6 (73.1, 86.7) | 97.8 (97.1, 98.3) | 0.73 (0.67, 0.78) |
| FCCr |  |  |  |  |  |  |  |
| 5+ | 235 | 9.5 | 96.4 (92.4, 98.7) | 96.8 (96.0, 97.5) | 68.9 (62.6, 74.8) | 99.7 (99.4, 99.9) | 0.79 (0.74, 0.83) |
| 6+ | 175 | 7.1 | 84.5 (78.2, 89.6) | 98.6 (98.0, 99.0) | 81.1 (74.5, 86.6) | 98.9 (98.3, 99.3) | 0.82 (0.77, 0.86) |
| 7+ | 143 | 5.8 | 78.0 (70.9, 84.0) | 99.5 (99.1, 99.7) | 91.6 (85.8, 95.6) | 98.4 (97.8, 98.9) | 0.83 (0.78, 0.88) |
| ***Sedatives (3.9%)*** |  |  |  |  |  |  |  |
| FC 3+ | 85 | 3.4 | 79.2 (69.7, 86.8) | 99.6 (99.3, 99.8) | 89.4 (80.8, 95.0) | 99.2 (98.7, 99.5) | 0.83 (0.77, 0.89) |
| FCr |  |  |  |  |  |  |  |
| 2+ | 113 | 4.6 | 93.8 (86.9, 97.7) | 99.0 (98.6, 99.4) | 79.6 (71.0, 86.6) | 99.7 (99.4, 99.9) | 0.86 (0.80, 0.91) |
| 3+ | 92 | 3.7 | 90.6 (82.9, 95.6) | 99.8 (99.5, 99.9) | 97.7 (96.4, 98.6) | 91.4 (90.0, 92.7) | 0.85 (0.83, 0.88) |
| 4+ | 87 | 3.5 | 90.6 (82.9, 95.6) | 100 (99.8, 100) | 100 (95.8, 100) | 99.6 (99.3, 99.8) | 0.95 (0.91, 0.98) |
| FCCr |  |  |  |  |  |  |  |
| 3+ | 104 | 4.2 | 99.0 (94.3, 100) | 99.6 (99.3, 99.8) | 91.3 (84.2, 96.0) | 100 (99.8, 100) | 0.95 (0.91, 0.98) |
| 4+ | 90 | 3.6 | 93.8 (86.9, 97.7) | 100 (99.8, 100) | 100 (96.0, 100) | 99.7 (99.5, 99.9) | 0.97 (0.94, 0.99) |
| 5+ | 88 | 3.6 | 91.7 (84.2, 96.3) | 100 (99.8, 100) | 100 (95.9, 100) | 99.7 (99.3, 99.9) | 0.96 (0.92, 0.98) |
| ***Prescription stimulants (3.8%)*** | |  |  |  |  |  |  |
| FC 3+ | 93 | 3.8 | 77.7 (67.9, 85.6) | 99.2 (98.7, 99.5) | 78.5 (68.8, 86.3) | 99.1 (98.7, 99.5) | 0.77 (0.70, 0.83) |
| FCr |  |  |  |  |  |  |  |
| 2+ | 124 | 5.0 | 89.4 (81.3, 94.8) | 98.3 (97.7, 98.8) | 67.7 (58.8, 75.9) | 99.6 (99.2, 99.8) | 0.76 (0.70, 0.82) |
| 3+ | 79 | 3.2 | 73.4 (63.3, 82.0) | 99.6 (99.2, 99.8) | 87.3 (78.0, 93.8) | 99.0 (98.5, 99.3) | 0.79 (0.72, 0.86) |
| 4+ | 68 | 2.7 | 72.3(62.2, 81.1) | 100 (99.8, 100) | 100 (94.7, 100) | 98.9 (98.4, 99.3) | 0.83 (0.76, 0.89) |
| FCCr |  |  |  |  |  |  |  |
| 3+ | 108 | 4.4 | 93.6 (86.6, 97.6) | 99.2 (98.7, 99.5) | 81.5 (72.9, 88.3) | 99.7 (99.4, 99.9) | 0.87 (0.81, 0.91) |
| 4+ | 77 | 3.1 | 81.9 (72.6, 89.1) | 100 (99.8, 100) | 100 (95.3, 100) | 99.3 (98.9, 99.6) | 0.90 (0.84, 0.94) |
| 5+ | 71 | 2.9 | 75.5 (65.6, 83.8) | 100 (99.8, 100) | 100 (94.9, 100) | 99.0 (98.6, 99.4) | 0.86 (0.79, 0.91) |
| ***Prescription painkillers (2.6%)*** | |  |  |  |  |  |  |
| FC 3+ | 47 | 1.9 | 64.6 (51.8. 76.1) | 99.8 (99.5, 99.9) | 89.4 (76.9, 96.5) | 99.1 (98.6, 99.4) | 0.74 (0.64, 0.83) |
| FCr |  |  |  |  |  |  |  |
| 2+ | 83 | 3.4 | 87.7 (77.2, 94.5) | 98.9 (98.4, 99.3) | 68.7 (57.6, 78.4) | 99.7 (99.3, 99.9) | 0.76 (0.68, 0.83) |
| 3+ | 51 | 2.1 | 75.4 (63.1, 85.2) | 99.9 (99.7, 100) | 96.1 (86.5, 99.5) | 99.3 (98.9, 99.6) | 0.84 (0.76, 0.91) |
| 4+ | 47 | 1.9 | 72.3 (59.8, 82.7) | 100 (9.8, 100) | 100 (92.5, 100) | 99.3 (98.8, 99.6) | 0.84 (0.76, 0.90) |
| FCCr |  |  |  |  |  |  |  |
| 3+ | 64 | 2.6 | 90.8 (81.0, 96.5) | 99.8 (99.5, 99.9) | 92.2 (82.7, 97.4) | 99.8 (99.5, 99.9) | 0.91 (0.86, 0.96) |
| 4+ | 53 | 2.1 | 81.5 (70.0, 90.1) | 100 (99.8, 100) | 100 (93.3, 100) | 99.5 (99.1, 99.7) | 0.90 (0.83, 0.95) |
| 5+ | 52 | 2.1 | 80.0 (68.2, 88.9) | 100 (99.8, 100) | 100 (93.2, 100) | 99.5 (99.1, 99.7) | 0.89 (0.82, 0.94) |

ASSIST = Alcohol, Smoking and Substance Involvement Screening Test; FC=frequency and concern; FCr=frequency and craving; FCCr=frequency, concern, and craving.

**Supplementary Table 3:** Prevalence of current use among those with ASSIST-FCCr binary versions

|  | **ASSIST-FCCr, 3+** | | | | | **ASSIST-FCCr, 4+** | | | | | **ASSIST-FCCr, 5+** | | | | |
| --- | --- | --- | --- | --- | --- | --- | --- | --- | --- | --- | --- | --- | --- | --- | --- |
|  |  | No current use | | With current use | |  | No current use | | With current use | |  | No current use | | With current use | |
|  | N with disorder | n | % | n | % | N with disorder | n | % | n | % | N with disorder | n | % | n | % |
| Tobacco | 977 | 178 | 18.2 | 799 | 81.8 | 785 | 4 | 0.5 | 781 | 99.5 | 776 | 4 | 0.5 | 772 | 99.5 |
| Alcohol^a^ | 442 | 0 | 0 | 442 | 100 | 353 | 0 | 0 | 353 | 100 | 204 | 0 | 0 | 204 | 100 |
| Cannabis | 319 | 52 | 16.3 | 267 | 83.7 | 239 | 3 | 1.3 | 236 | 98.7 | 235 | 3 | 1.3 | 232 | 98.7 |
| Sedatives | 104 | 9 | 8.7 | 95 | 91.3 | 90 | 0 | 0 | 90 | 100 | 88 | 0 | 0 | 88 | 100 |
| Prescription stimulants | 108 | 20 | 18.5 | 88 | 81.5 | 77 | 0 | 0 | 77 | 100 | 71 | 0 | 0 | 71 | 100 |
| Prescription painkillers | 64 | 10 | 15.6 | 54 | 84.8 | 53 | 1 | 1.9 | 52 | 98.1 | 52 | 1 | 1.9 | 51 | 98.1 |

ASSIST = Alcohol, Smoking and Substance Involvement Screening Test; FCCr=frequency, concern, and craving

a thresholds are 8+, 9+, 10+
